# Antibiotics against *Pseudomonas aeruginosa* on Human Skin Cell Lines: Determination of the Highest Non-Cytotoxic Concentrations with Antibiofilm Capacity for Wound Healing Strategies

**DOI:** 10.3390/pharmaceutics16010117

**Published:** 2024-01-17

**Authors:** María I. Quiñones-Vico, Ana Fernández-González, Ana Ubago-Rodríguez, Kirsten Moll, Anna Norrby-Teglund, Mattias Svensson, José Gutiérrez-Fernández, Jesús M. Torres, Salvador Arias-Santiago

**Affiliations:** 1 Cell Production and Tissue Engineering Unit, Virgen de las Nieves University Hospital, 18014 Granada, Spain; maribelqv@ugr.es (M.I.Q.-V.); aur@ugr.es (A.U.-R.); salvadorarias@ugr.es (S.A.-S.); 2 Biosanitary Institute of Granada (ibs.GRANADA), 18014 Granada, Spain; 3 Andalusian Network of Design and Translation of Advanced Therapies, 41092 Seville, Spain; 4 Dermatology Department, School of Medicine, University of Granada, 18016 Granada, Spain; 5 Biochemistry, Molecular Biology III and Immunology Department, University of Granada, 18071 Granada, Spain; torrespi@ugr.es; 6 Center for Infectious Medicine, Karolinska Institutet, Karolinska University Hospital Huddinge, 141 86 Stockholm, Sweden; kirsten.moll@ki.se (K.M.); anna.norrby-teglund@ki.se (A.N.-T.); mattias.svensson@ki.se (M.S.); 7 Microbiology Department, Virgen de las Nieves University Hospital, 18014 Granada, Spain; josegf@ugr.es; 8 Dermatology Department, Virgen de las Nieves University Hospital, 18014 Granada, Spain

**Keywords:** antibiotics, biofilm, fibroblasts, keratinocytes, *Pseudomonas aeruginosa*, wound healing

## Abstract

*Pseudomonas aeruginosa* is one of the most common microorganisms causing infections of severe skin wounds. Antibiotic or antiseptic treatments are crucial to prevent and curb these infections. Antiseptics have been reported to be cytotoxic to skin cells and few studies evaluate the impact of commonly used antibiotics. This study evaluates how clinical antibiotics affect skin cells’ viability, proliferation, migration, and cytokine secretion and defines the highest non-cytotoxic concentrations that maintain antibacterial activity. Cell proliferation, viability, and migration were evaluated on cell monolayers. Cytokines related to the wound healing process were determined. The minimum inhibitory concentrations and the impact on bacterial biofilm were assessed. Results showed that 0.02 mg/mL ciprofloxacin and 1 mg/mL meropenem are the highest non-cytotoxic concentrations for fibroblasts and keratinocytes while 1.25 mg/mL amikacin and 0.034 mg/mL colistin do not affect fibroblasts’ viability and cytokine secretion but have an impact on keratinocytes. These concentrations are above the minimum inhibitory concentration but only amikacin could eradicate the biofilm. For the other antibiotics, cytotoxic concentrations are needed to eradicate the biofilm. Combinations with colistin at non-cytotoxic concentrations effectively eliminate the biofilm. These results provide information about the concentrations required when administering topical antibiotic treatments on skin lesions, and how these antibiotics affect wound management therapies. This study set the basis for the development of novel antibacterial wound healing strategies such as antibiotic artificial skin substitutes.

## 1. Introduction

Skin is the largest organ of the human body and protection is one of its most important functions. It constitutes a protective barrier against the invasion of microorganisms and other aggressive external factors including mechanical, chemical, thermal, and osmotic agents. Other crucial functions are the regulation of body temperature and the power of regeneration thanks to its self-healing capacity [1,2,3]. 

Loss of skin integrity due to injury or illness can cause physiologic imbalance and ultimately significant disability or even death. Acute trauma, chronic wounds, infections, surgical interventions, and genetic disorders are the most common causes of skin injury [1]. In the case of severe injuries, the self-healing capacity of the skin is insufficient and the wound-healing process can be compromised, leaving the body open to infection and poor thermal and fluid regulation [4]. Therefore, approaches to help and improve wound healing are needed. 

Traditional approaches for wound management include autologous skin grafts and wound dressings. However, research is focused on developing new approaches to be used in combination or as a replacement for traditional management. Among these new strategies, the administration of autologous skin cell suspension and the development of skin substitutes through tissue engineering techniques are highlighted [1,4,5,6]. Both approaches are mainly composed of human fibroblasts (HFs) and human keratinocytes (hKTs), which are the predominant cell types in the skin. Furthermore, these cells are involved in the wound-healing process [4].

In patients with severe injuries, antibiotic or antiseptic treatments are crucial to prevent and curb possible infections that may occur. *Pseudomonas aeruginosa* (*P. aeruginosa*) is one of the most common microorganisms causing infections of severe skin wounds, burns, and chronic skin ulcers [7,8]. The cutaneous manifestations of these infections are highly variable, ranging from mild, self-limiting syndromes to life-threatening diseases [7]. Biofilms, which are complex clusters of bacteria attached to a surface and embedded in a self-produced matrix, lead to an organized microbial ecosystem in which bacteria develop antibiotic tolerance [9]. In fact, bacteria entrapped in biofilms can be up to 1000-fold more tolerant to antibiotics than free-living bacteria [10]. Thus, biofilms significantly delay wound healing and lead to a chronic inflammatory state, disrupting the wound-healing process [11].

Antiseptics have been reported to be cytotoxic to skin cell lines and skin substitutes at clinical concentrations [12,13,14]. However, few studies in the literature evaluate the impact of commonly used antibiotics on these cells and skin substitutes. Furthermore, the incidence of multi-drug-resistant (MDR) strains of *P. aeruginosa* has been steadily increasing and the use of broad-spectrum antimicrobials has been linked to this increment and to the emergence of antimicrobial resistance [7]. 

To overcome the cytotoxicity of antiseptics and the antimicrobial resistance to commonly used antibiotics, research efforts are focusing on developing a new generation of biological bandages with antimicrobial properties to treat bacterial infections topically. For instance, several approaches focus on the development of antibiotic-loaded nanoparticles to be applied at the site of infection [15], antibiotic-loaded polymeric composites and dressings [16,17], or hydrogels [18]. Another interesting strategy is the creation of skin substitutes with antimicrobial capacity by the incorporation of nanoparticulate systems containing antibiotics. Since the main cellular components of these skin substitutes are HFs and hKTs, the assessment of the impact of commonly used antibiotics on skin cell viability is necessary to determine the non-cytotoxic concentrations for these cells. Furthermore, as *P. aeruginosa*’s biofilms represent a significant and threatening problem for skin-injured patients, it is crucial to evaluate how antibiotics affect and eradicate these biofilms. 

Specifically, the objective of this study is to evaluate how amikacin (AMK), ciprofloxacin (CIP), colistin (COL), and meropenem (MER) affect viability, proliferation, migration, wound closure, and cytokine secretion of HFs and hKTs in order to establish the highest concentrations of these antibiotics, which are not cytotoxic to these cells but have an antimicrobial activity to *P. aeruginosa*, including also biofilm. 

## 2. Materials and Methods

### 2.1. Cell Isolation

HFs were obtained from 9 cm^2^ skin samples taken during dermatological surgery with the patient’s prior consent, following the regulations for human cell and tissue donation (Royal Decree-Law 9/2014, of 4 July). 

The skin samples were processed as previously reported [14]. Briefly, the dermis and the epidermis were mechanically separated. The dermis was incubated in a 2 mg/mL solution of type I collagenase (Gibco, Thermo Fisher Scientific, Carlsbad, CA, USA) for 18–24 h. Türk (Sigma Aldrich, St. Louis, MO, USA) and Trypan Blue (Sigma Aldrich, St. Louis, MO, USA) solutions were used to evaluate cell counting and viability, respectively. Fibroblasts were initially seeded at a density of 100,000–140,000 cells/cm^2^ and at 5000–7000 cells/cm^2^ after passaging.

The HaCaT cell line, which is a human keratinocyte cell line (CLS Cell Lines Service, 300493), was utilized as a model to investigate the cytotoxicity of keratinocytes. HaCaT cells were seeded at a density of 10,000 cells/cm^2^.

### 2.2. Evaluation of Antibiotic Treatment

#### 2.2.1. Cells and Antibiotics

HFs and hKTs were seeded at a density of 7500 cells/cm^2^ in 24-well plates (Thermo Fisher Scientific, Carlsbad, CA, USA). The culture medium was supplemented with antibiotics effective against *P. aeruginosa*. This culture medium was changed every 72 h. The different evaluations were performed at days 3, 7, 10, and 14 of culture. The antibiotics used were AMK (Normon Laboratories, Madrid, Spain), CIP (Fresenius Kabi España, Barcelona, Spain), COL (Altan Pharmaceuticals, Madrid, Spain), and MER (Aurovitas Spain, Madrid, Spain), all approved for clinical use. The culture medium for the control was not supplemented.

A preliminary evaluation was carried out to determine which was the highest concentration of antibiotic where cell proliferation was unaltered (Figures S1 and S2). The concentrations chosen for the respective antibiotics and the two cell types are listed in Table 1. Each antibiotic was used in triplicate (*n* = 3) in the different assays described in Section 2.2.2, Section 2.2.3, Section 2.2.4 and Section 2.2.5.

#### 2.2.2. Cell Proliferation Assay

AlamarBlue™ HS Cell Viability Reagent (Invitrogen by Thermo Fisher Scientific, Carlsbad, CA, USA) was used to determine cell proliferation. This reagent is a pre-made resazurin-based solution that enables the quantification of cell proliferation based on the reducing capacity of living cells. The reagent was added to each well at a volume of 10% and then incubated in the dark at a temperature of 37 °C with a CO_2_ concentration of 5% for four hours. The fluorescence was measured at a wavelength of 560/590 nm using a 96-well plate. Cell proliferation was determined by the reduction in the reagent and the density of live cells, by using a standard curve.

#### 2.2.3. Cell Viability Assay

Cell viability was assessed using the Live/Dead^®^ cell viability assay (Thermo Fisher Scientific, Carlsbad, CA, USA). This colorimetric assay involves preparing a staining solution that includes calcein AM (green fluorescence, Ex/Em 494/517 nm) and ethidium homodimer-1 (red fluorescence, Ex/Em 517/617 nm). This allows the differentiation of live cells, which will be stained green from dead cells, which will be stained red. The Live/Dead^®^ solution was applied and incubated for 30 min in the dark at room temperature. Following the incubation period, the solution was extracted and rinsed. Fluorescence was then assessed at a wavelength of 405 nm using a Leica DM2000 microscope (Leica, Wetzlar, Germany).

#### 2.2.4. Wound Closure Assay

HFs and hKTs were seeded in 12-well plates (Thermo Fisher Scientific, Carlsbad, CA, USA) instead of 24-well plates, at a density of 7500 cells/cm^2^. Confluent cells were scratched to simulate wound formation, followed by incubation in antibiotic-supplemented media at the concentrations described in Table 1. Control cells were untreated. Images were captured of the scraped area in each well until the control was fully closed.

ImageJ2 software was used to analyze the images [19]. The percentage of wound closure and the rate of cell migration (µm/h) were calculated using Equations (1) and (2), respectively [13]. The percentage of wound closure in the scratched area was calculated at 6, 12, 15, and 22 h for HFs and at 12, 24, 30, and 34 h for hKTs. Wound closure was monitored until complete closure of the control was achieved in each cell line.

(1) $$\;Wound\;closure\;\left(\%\right)=\left(\frac{A_{t=0}-A_{t=t}}{A_{t=0}}\right)\times 100$$

(2) $$Cell\;migration\;rate\;\left(\frac{µm}{h}\right)=\left(\frac{W_{i}-W_{f}}{t}\right)$$

where “*A*_*t*=0_” represents the initial area of the wound immediately after scratching, “*A*_*t*=*t*_” represents the area of the wound after “n” hours of initial scratching, “*W_i_*” represents the average width of the initial wound in µm, “*W_f_*” represents the average width of the final wound in µm, and “*t*” represents the duration of the assay (in hours) until the control wound is completely closed.

#### 2.2.5. Cytokine Evaluation

The levels of interleukin 10 (IL-10, Sigma Aldrich, St. Louis, MO, USA), basic fibroblast growth factor (bFGF, Sigma Aldrich, St. Louis, MO, USA), tumor necrosis factor alpha (TNF-α, Sigma Aldrich, St. Louis, MO, USA) and interleukin 6 (IL-6, Sigma Aldrich, St. Louis, MO, USA) were quantified in cell supernatants at days 3, 7, 10, and 14 by an ELISA assay according to the manufacturer’s instructions. The absorbance was measured at 450 nm, and the protein concentration was determined using a standard plot.

### 2.3. Determination of the Antibacterial Capacity

#### 2.3.1. Minimum Inhibitory Concentration (MIC)

The susceptibility study was conducted by broth microdilution using the automated MicroScan Walkaway system (Beckman-Coulter, Brea, CA, USA) and diffusion gradient strips (MIC Test Strip, Liofilchem, Italy) for *P. aeruginosa* ATCC 27853, as recommended by the European Committee on Antimicrobial Susceptibility Testing (EUCAST) [20]. The MIC of colistin was determined using broth microdilution, following the recommendations of the CLSI documents M07-A8 and M100-S31 [21,22]. We used a concentration range between 0.5 and 8 μg/mL colistin sulfate analytical grade (Sigma-Aldrich. Code C2700000.Batch 3.0, St. Louis, MO, USA) and Mueller Hinton broth with cation adjustment (Thermo Fisher Scientific, Carlsbad, CA, USA).

#### 2.3.2. Antibiofilm Capacity

- Surface-attached biofilms preparation

Surface-attached biofilms on HF monolayers were prepared by seeding HFs (2 × 10^4^) in plastic inserts (activated calWells; Symcel, Sweden) and growing them at 37 °C, 5% CO_2_ in EpiLife medium (Invitrogen). At 70% confluence, 4 µL/insert of 1/100 bacterial dilution was inoculated and allowed to establish biofilm for 24 h. Biofilm formation was indicated by bacterial aggregates using light microscopy. At 24 h and 48 h, EpiLife was replaced with medium containing different concentrations of AMK, CIP, COL, and MER. After that, the medium was replaced with Mueller–Hinton broth, and microcalorimetric measurements were performed.

- Microcalorimetry assay

Microcalorimetric measurements were carried out using the CalScreener according to the manufacturer’s instructions (Symcel AB, Stockholm, Sweden) as previously reported [23,24]. CalWells with prepared samples (32×) and thermodynamic references (16×) were placed into sterile titanium vials and sealed with individual lids tightened to 40 cNm torque. The samples were heated and allowed to reach equilibrium in two steps, with the first step lasting 10 min and the second step lasting 20 min. After equilibration for 1 to 2 h, the heat flow (mW) per insert was measured every 42 s for a total of 120 h. Results were analyzed using calView and calData software (Symcel AB, Stockholm, Sweden).

### 2.4. Statistical Analysis

The data were analyzed using GraphPad Prism 9.0 Software, Inc. (La Jolla, CA, USA) for statistical analysis. The results are presented as the mean ± standard error of the mean (SEM). The analysis involved applying a factorial analysis of variance (ANOVA) to determine the effect of each factor. After conducting the ANOVA test, a post hoc analysis using Tukey’s test was performed on all factors to assess the level of significance in comparing the factor classes. A *p*-value of ≤0.05 was considered to indicate statistical significance.

## 3. Results

### 3.1. HFs’ Proliferation and Viability Are Intact after Antibiotic Treatments

**Figure 1:**
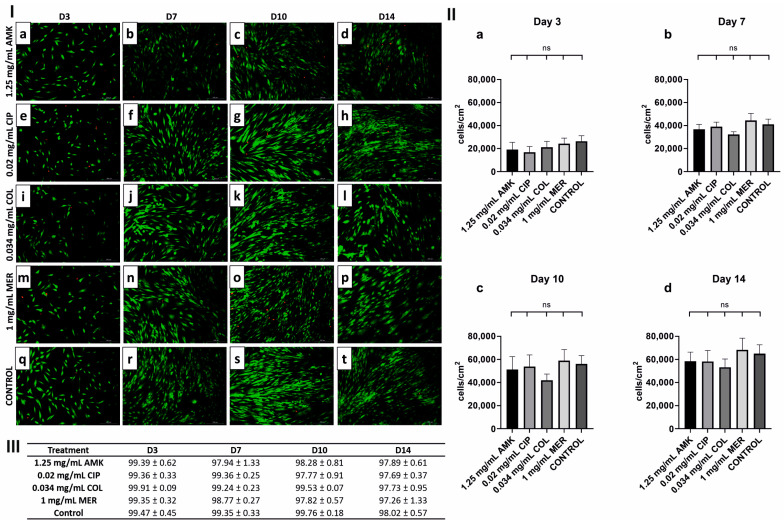
Cell viability and proliferation in HFs after antibiotic treatment on days 3, 7, 10, and 14. (**I**) Viability of HFs after each treatment and the control on days 3, 7, 10, and 14. (**a**–**d**) LIVE/DEAD^®^ images of HFs after AMK (1.25 mg/mL) treatment on days 3, 7, 10, and 14, respectively. (**e**–**h**) HFs after CIP (0.02 mg/mL) treatment on days 3, 7, 10, and 14, respectively. (**i**–**l**) HFs after COL (0.034 mg/mL) treatment on days 3, 7, 10, and 14, respectively. (**m**–**p**) HFs after MER (1 mg/mL) treatment on days 3, 7, 10, and 14, respectively. (**q**–**t**) Control (without treatment) on days 3, 7, 10, and 14, respectively. Dead cells are shown in red and live cells in green. Representative images are shown; *n* = 3 experiments. Magnification 10×. (**II**) Bar graph showing the cell density in HFs on (**a**) day 3, (**b**) day 7, (**c**) day 10, and (**d**) day 14 of treatment. ns: No statistically significant differences were observed. (**III**) Cell viability percentage of HFs after treatments. AMK, amikacin; CIP, ciprofloxacin; COL, colistin; MER, meropenem. *n* = 3 experiments. The absence of an asterisk indicates statistically non-significant differences as compared to the control group, *p* > 0.05.

Considering the results of the preliminary study (Figures S1 and S2), HFs’ proliferation and viability were evaluated after 1.25 mg/mL AMK, 0.02 mg/mL CIP, 0.034 mg/mL COL and 1 mg/mL MER treatments (*n* = 3). All treatments resulted in a cell proliferation similar to the control and no significant differences were observed at days 3, 7, 10, and 14 of follow-up (Figure 1I). Cell viability was also not affected (Figure 1II–III). 

### 3.2. hKTs’ Proliferation and Viability Are Affected after AMK and COL Treatments

**Figure 2:**
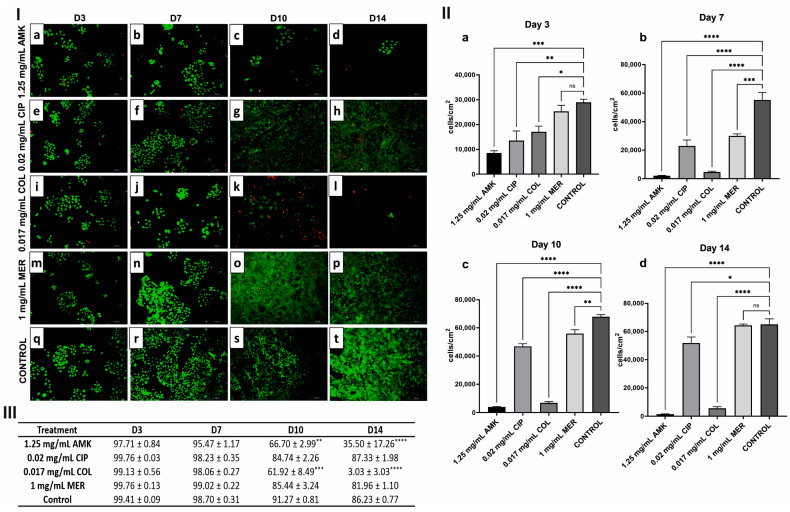
Cell viability and proliferation in hKTs after antibiotic treatment on days 3, 7, 10, and 14. (**I**) Viability of hKTs after each treatment and the control on days 3, 7, 10, and 14. (**a**–**d**) LIVE/DEAD^®^ images of hKTs after AMK (1.25 mg/mL) treatment on days 3, 7, 10, and 14, respectively. (**e**–**h**) hKTs after CIP (0.02 mg/mL) treatment on days 3, 7, 10, and 14, respectively. (**i**–**l**) hKTs after COL (0.017 mg/mL) treatment on days 3, 7, 10, and 14, respectively. (**m**–**p**) hKTs after MER (1 mg/mL) treatment on days 3, 7, 10, and 14, respectively. (**q**–**t**) Control (without treatment) on days 3, 7, 10, and 14, respectively. Dead cells are shown in red and live cells in green. Representative images are shown; *n* = 3 experiments. Magnification 10×. (**II**) Bar graph showing the cell density in hKTs on (**a**) day 3, (**b**) day 7, (**c**) day 10, and (**d**) day 14 of treatment. ns: No statistically significant differences were observed. (**III**) Cell viability percentage of hKTs after treatments at days 3, 7, 10, and 14. AMK, amikacin; CIP, ciprofloxacin; COL, colistin; MER, meropenem. *n* = 3 experiments. * *p* ≤ 0.05; ** *p* ≤ 0.004; *** *p* ≤ 0.0007; **** *p* < 0.0001. The absence of an asterisk indicates non-significant differences as compared to the control group, *p* > 0.05.

In hKTs, there were significant differences in cell density based on the treatment administered and compared to the control (Figure 2I). At day 3, the proliferation of hKTs was significantly decreased following the administration of all antibiotics except for 1 mg/mL MER. At day 7, all treatments caused a significant reduction in cell density compared to the control. However, for AMK and COL, this reduction was greater. At day 10, cell density after CIP and MER was recovered although it was still significantly different from the control. However, at day 14, these differences were reduced and cell density after CIP and MER were similar to the control. Cell density after AMK and COL treatments was not recovered. 

Cell viability was affected after AMK and COL after 14 days of follow-up compared to CIP and MER and control (Figure 2II–III). 

### 3.3. HFs’ and hKTs’ Wound Closure and Migration Are Intact after Antibiotic Treatments

**Figure 3:**
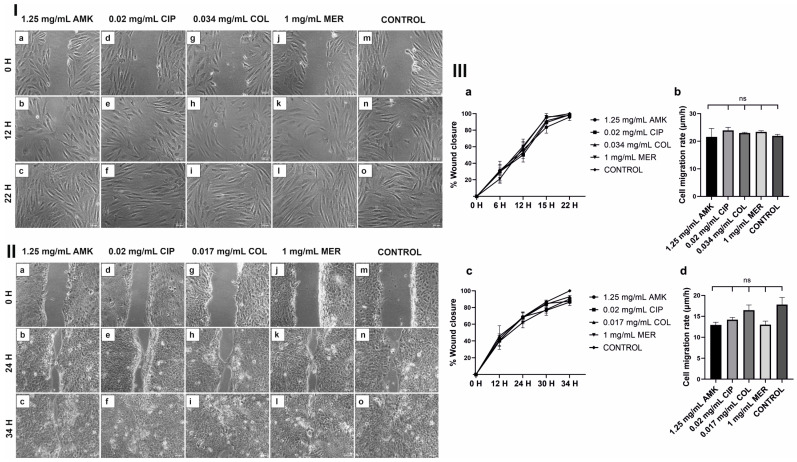
Wound closure in HFs and hKTs after antibiotic treatments. Most representative images are shown. For additional information see Figures S3 and S4. (**I**) (**a**–**c**) The wound closure assay was performed on HFs after treatment with 1.25 mg/mL AMK at 0, 12, and 22 h after scratching, respectively. (**d**–**f**) The HFs were treated with 0.02 mg/mL CIP at 0, 12, and 22 h after scratching, respectively. (**g**–**i**) The HFs were treated with 0.034 mg/mL COL at 0, 12, and 22 h after scratching, respectively. (**j**–**l**) The HFs were treated with 1 mg/mL MER at 0, 12, and 22 h after scratching, respectively. (**m**–**o**) The control group was observed at 0, 12, and 22 h after scratching, respectively. *n* = 3. Magnification 10×. (**II**) (**a**–**c**) The wound closure assay was performed on hKTs after treatment with 1.25 mg/mL AMK at 0, 24, and 34 h after scratching, respectively. (**d**–**f**) hKTs were treated with 0.02 mg/mL CIP at 0, 24, and 34 h after scratching, respectively. (**g**–**i**) hKTs were treated with 0.017 mg/mL COL at 0, 24, and 34 h after scratching, respectively. (**j**–**l**) hKTs were treated with 1 mg/mL MER at 0, 24, and 34 h after scratching, respectively. (**m**–**o**) Control samples were taken at 0, 24, and 34 h after scratching, respectively. *n* = 3. Magnification 10×. (**III**) Wound closure and cell migration rate in HFs and hKTs after antibiotic treatments. (**a**) Percentage of wound closure ± SEM in HFs at 0, 6, 12, 15, and 22 h. (**b**) Bar graph of average HFs migration rate (µm/h) at 0, 6, 12, 15, and 22 h. (**c**) Percentage of wound closure ± SEM in hKTs at 0, 12, 24, 30, and 34 h. (**d**) Bar graph of average hKTs migration rate (µm/h) at 0, 12, 24, 30, and 34 h. ns: no significant differences.

Cell migration in HFs and hKTs was not affected by the antibiotic treatments compared to the control. As shown in Figure 3I, 22 h after treatments all wounds had closed in HF monolayers similarly to the control. For hKTs monolayers, all wounds closed 34 h after treatments (Figure 3II). 

Regarding wound closure percentages and cell migration rates (Figure 3III), no significant differences were observed between antibiotic treatments and control.

### 3.4. The Secretion of Cytokines Involved in Wound Healing Is Not Altered in HFs but Is Affected in hKTs

**Figure 4:**
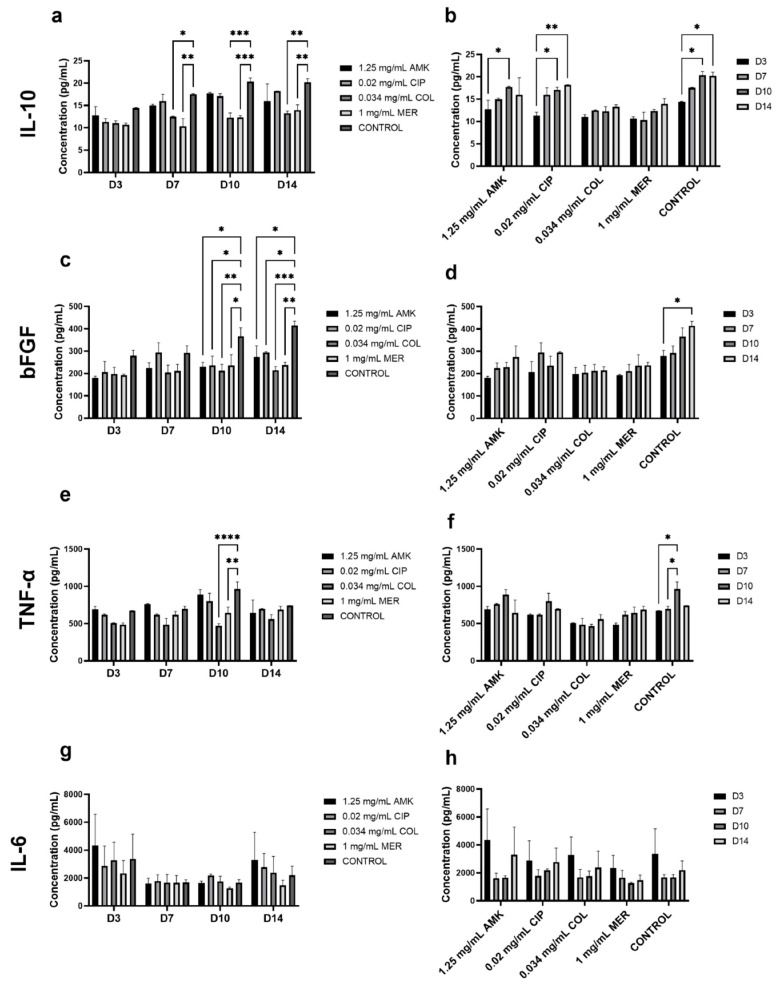
Cytokine concentrations in HFs supernatant on days 3, 7, 10, and 14. Corresponding to IL-10 (**a**,**b**), bFGF (**c**,**d**), TNF-α (**e**,**f**), and IL-6 (**g**,**h**). *n* = 3. * *p* ≤ 0.05; ** *p* ≤ 0.01; *** *p* ≤ 0.001; **** *p* ≤ 0.0001.

The concentration of IL-10, bFGF, TNF-α, and IL-6 was measured in HF supernatants at days 3, 7, 10, and 14 after antibiotic treatment (Figure 4, Tables S1–S4). 

Regarding IL-10 analysis, no significant differences were observed between antibiotic treatments and control at day 3. At days 7, 10, and 14, the concentration of IL-10 was significantly reduced after COL and MER compared to the control. However, after all antibiotic treatments, the concentration of IL-10 was increasing as the days of study passed in a similar way to the control. The levels of bFGF concentration were significantly elevated in the control group compared with all antibiotics at days 10 and 14 but a progressive increase in this concentration is observed in all groups. TNF-α concentration was significantly reduced after COL and MER treatments when compared to the control at day 10. With respect to the progression over time, significant differences were observed in the control group where the concentration of this cytokine was higher at day 10 compared to days 3 and 7. In the IL-6 analysis, no significant differences were observed between antibiotic treatment and the control. Furthermore, no significant differences were observed between the days of follow-up. 

**Figure 5:**
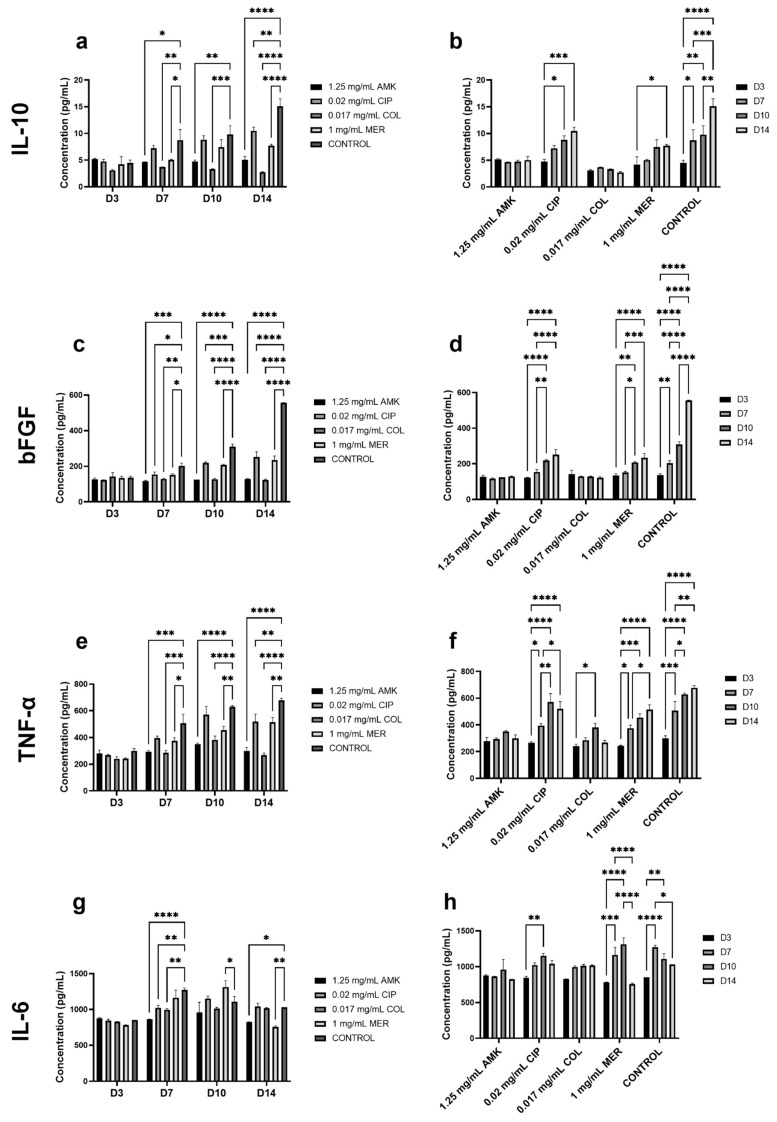
Cytokine concentrations in hKTs supernatant on days 3, 7, 10, and 14. Corresponding to IL-10 (**a**,**b**), bFGF (**c**,**d**), TNF-α (**e**,**f**), and IL-6 (**g**,**h**). *n* = 3. * *p* ≤ 0.05; ** *p* ≤ 0.01; *** *p* ≤ 0.001; **** *p* ≤ 0.0001.

IL-10, bFGF, TNF-α, and IL-6 were also measured in hKTs supernatants at days 3, 7, 10, and 14 (Figure 5, Tables S5–S8).

Regarding IL-10 analysis, at days 7, 10, and 14, the secretion was significantly reduced after AMK and COL compared to the control. At days 7 and 14, MER treatment also reduced IL-10 concentration. This reduction was less pronounced than after AMK and COL. The concentration of IL-10 increases with time after CIP and MER as in the control group. After AMK and COL treatments, no significant differences were observed regarding the follow-up time. A similar pattern to IL-10 is observed for bFGF. At days 7, 10, and 14, all antibiotic treatments significantly reduced bFGF secretion when compared to the control. However, these reductions were higher after AMK and COL. Furthermore, the secretion of bFGF increased significantly with time after CIP and MER treatments and in the control group but no significant differences were observed after AMK and COL treatments. TNF-α secretion, at days 7 and 10, was significantly reduced after AMK, COL, and MER treatments when compared to the control. At day 14, all antibiotic treatments significantly reduced TNF-α concentration. These reductions were more accentuated after AMK and COL treatments. A similar progression to control was observed in the secretion of this cytokine after treatment CIP and MER treatments compared to AMK and COL treatments. The level of IL-6 concentration was significantly increased in the control group with respect to AMK, CIP, and COL at day 7. At day 10, MER significantly increased the IL-6 level compared to the control. But, at day 14, this treatment significantly reduced IL-6 concentration. AMK also reduced this concentration when compared to the control. Regarding the secretion pattern through time, significant increases in the concentration of this cytokine were observed at days 7 and 10 with respect to days 3 and 14 after CIP and MER treatments and in the control group. However, no significant differences were observed regarding AMK and COL treatments. 

### 3.5. Antimicrobial Activity of Antibiotic Treatments against P. aeruginosa

MIC values for AMK, CIP, COL, and MER against *P. aeruginosa* ATCC 27853 are shown in Table 2. Hence, the selected highest non-cytotoxic concentrations, i.e., 1.25 mg/mL AMK, 0.02 mg/mL CIP, 0.017 mg/mL COL, and 1 mg/mL MER, are all above the MIC values for each antibiotic. 

### 3.6. Impact of Antibiotic Treatments on P. aeruginosa’s Biofilm

**Figure 6:**
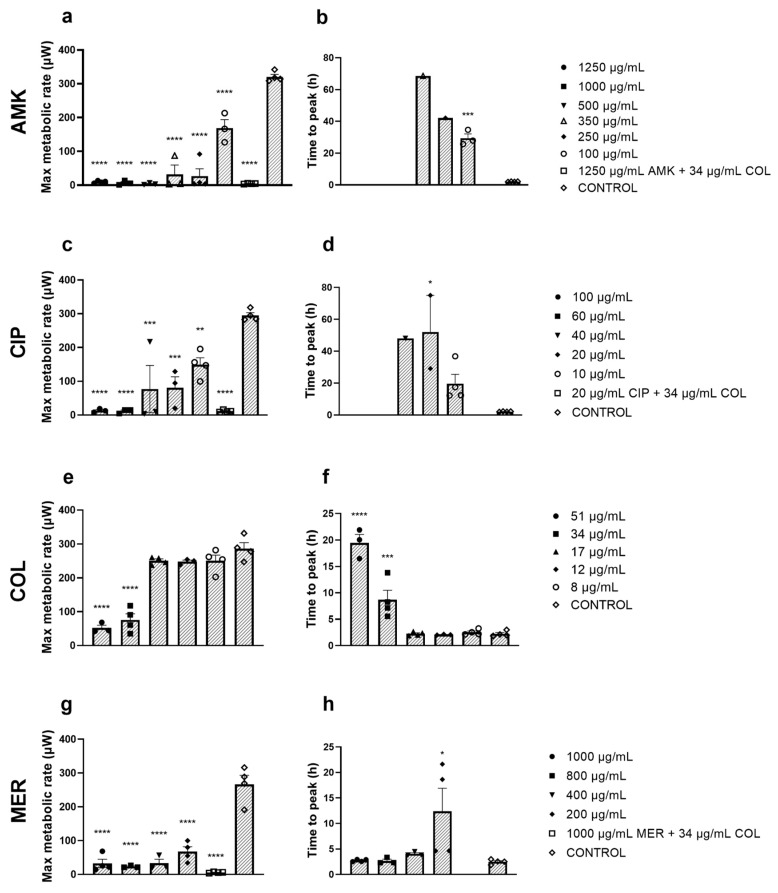
Real-time calorimetric measurement of *P. aeruginosa*-infected HFs after AMK treatment (**a**,**b**), CIP treatment (**c**,**d**), COL treatment (**e**,**f**), and MER treatment (**g**,**h**). Controls are included in each condition. Combinations of every antibiotic with COL are included in the corresponding graph; (**a**,**b**) for AMK–COL combination, (**c**,**d**) for CIP–COL combination, and (**g**,**h**) for MER–COL combination. *n* = 3. AMK, amikacin; CIP, ciprofloxacin; COL, colistin; MER, meropenem. * *p* ≤ 0.05; ** *p* ≤ 0.03; *** *p* ≤ 0.008; **** *p* ≤ 0.0001. Absence of an asterisk means no significant difference.

To assess the impact of antibiotics on *P. aeruginosa*’s biofilm on HF monolayer, microcalorimetry was used to monitor maximum metabolic rate and lag extension time (i.e., time to peak) after antibiotic treatments [25]. Regarding AMK, the 1250 µg/mL–500 µg/mL range effectively eradicated the biofilm as no growth was observed after treatments (Figure 6a,b). The other concentrations (350–100 µg/mL) were less efficient in their antimicrobial efficacy, as shown by both the maximum metabolic rate and the time to peak. 

For CIP, COL, and MER treatments, the highest non-cytotoxic concentrations for cells were unable to eliminate the biofilm as bacterial growth was observed. However, the metabolic rate in these cases was significantly lower compared to the control (Figure 6c,e,g). Regarding lag extension time, except for MER, there is a decreasing trend when reducing antibiotic concentration (Figure 6d,f,h). Importantly, combinations of the highest non-cytotoxic concentrations of every antibiotic with colistin were effective in eradicating the biofilm as no growth was noticed (Figure 6).

## 4. Discussion and Conclusions

Antibiotics have provided protection against life-threatening bacterial infections, and they are crucial to control *P. aeruginosa* infection in patients with severe skin injuries. This bacterium is well-known for causing various skin conditions, some of which can be severe and potentially fatal [26]. Nevertheless, the emergence of MDR organisms has been caused by the excessive use of antibiotics and the evolution of organisms [27]. New approaches for wound healing providing antibiotic capacity are needed. HFs and hKTs are part of wound healing strategies including the administration of autologous skin cell suspensions [28,29,30] and the grafting of skin substitutes [3,5] to the wound site. Since the administration of antibiotics is necessary to control and prevent infections, it is essential to assess how these antibiotics affect skin cells and to determine the highest non-cytotoxic concentrations of these antibiotics with antibacterial and antibiofilm capacity. Thus, the study is built in two phases: (1) determining the highest non-cytotoxic concentrations for skin cells and (2) determining if those concentrations are effective against a *P. aeruginosa* infection model. 

In this study, a range of concentrations of AMK, CIP, COL, and MER were tested in the skin cell lines. AMK is a semi-synthetic antibiotic derived from kanamycin with strong activity against Gram-negative bacteria. It can still fight off bacteria even when exposed to enzymes that typically break down other aminoglycosides [31,32]. CIP is a quinolone with a broad spectrum of effects on Gram-negative bacteria, which acts by inhibiting the DNA gyrase enzyme [33]. COL is a polymyxin with strong electrostatic interaction with bacterial membranes [34] and MER is a broad-spectrum beta-lactam. It works by stopping the synthesis of peptidoglycan, which leads to the death of bacterial cell walls [35].

According to our results, concentrations above 1.25 mg/mL AMK, 0.02 mg/mL CIP, 0.034 mg/mL COL, and 1 mg/mL MER affect HFs proliferation. For hKTs, cell proliferation is affected by concentrations above 1.25 mg/mL AMK, 0.02 mg/mL CIP, 0.017 mg/mL COL, and 1 mg/mL MER. Therefore, these concentrations were considered as the highest non-cytotoxic for these cells, and cell viability, cell migration, and cytokine secretion after treatments with these concentrations were evaluated in triplicate. 

Regarding HFs, 1.25 mg/mL AMK, 0.02 mg/mL CIP, 0.034 mg/mL COL, and 1 mg/mL MER did not affect cell proliferation, viability, migration, and wound closure as compared to the control. Concerning hKTs, 1.25 mg/mL AMK and 0.017 mg/mL COL significantly reduced cell proliferation as compared to CIP and MER treatments and control at days 7, 10, and 14. Regarding cell viability, AMK and COL had a greater impact than CIP and MER at days 10 and 14. At day 7, hKTs viability was not affected. The different results of cell proliferation and viability at day 7 may be because, although the hKTs are viable, their proliferative capacity and metabolic activity are affected. No significant differences were observed regarding cell migration and wound closure. All wounds closed after 34 h. This is consistent with the fact that the viability and proliferation of the cells decrease after day 3 of the study. Therefore, AMK and COL have a greater impact on hKTs than on HFs. CIP and MER do not present cytotoxicity for any of these cell lines. These results agree with the study carried out by Damour et al. where they showed that AMK and COL were less cytotoxic for HFs than for hKTs since the CD50 was much higher in the first case [36]. Seabra-Souza et al. also evaluated the cytotoxicity of AMK on HFs and found that concentrations ranging from 0.011 to 0.033 mg/mL did not affect the cellular viability [37]. Regarding CIP treatment, Gürbay et al. found that concentrations above 0.129 mM (0.04 mg/mL) caused a significant level of cytotoxicity on HFs following 72 h of exposure [38] and Ferreira et al. showed that concentrations below 0.05 mg/mL preserved HFs viability [39]. These results are consistent with what was observed in our study. Regarding the responsible mechanisms for the observed cytotoxic effects, Prokhorova et al. reported that the cytotoxicity of AMK to eukaryotic cells may be due to the interaction of this antibiotic with protein synthesis at the molecular level [40]. Their study showed that aminoglycosides interact with eukaryotic 80S ribosome inhibiting the intersubunit movement. This is supported by Seely et al., who demonstrated that AMK disrupts the process of transfer RNA (tRNA) translocation, release factor-mediated peptidyl-tRNA hydrolysis, and ribosome recycling [41]. Regarding COL, its cytotoxicity to eukaryotic cells may be related to its effect on lipid membranes, particularly its effects on mitochondria in eukaryotic cells [42]. In this study, the cytotoxicity of the antibiotics was found to be higher for hKTs than for HFs. This difference may be related to the higher metabolic activity and proliferation of hKTs, leading to increased protein synthesis and mitochondrial activity based on information regarding molecular mechanisms. No study to date has evaluated the impact of MER on HFs and hKTs. 

There are no studies in the literature evaluating the secretion of cytokines related to the inflammatory and wound-healing process after antibiotic treatments. IL-10 is a cytokine that has anti-inflammatory properties and plays a role in promoting the healing of epithelial wounds without scarring [43,44]. bFGF stimulates angiogenesis, fibroblast proliferation, and infiltration, and promotes epithelial cell migration and proliferation [45]. TNF-α is a cytokine that promotes inflammation and wound healing. It can stimulate inflammation and increase the production of growth factors by macrophages, which aids in wound healing. However, at higher levels, it can hinder cell migration by suppressing the synthesis of extracellular matrix and increasing the synthesis of metalloproteinases [45,46]. IL-6 stimulates the release of proinflammatory cytokines from macrophages, keratinocytes, endothelial cells, and stromal cells in the tissues. As inflammation advances, IL-6 signaling is accountable for the transition to a reparative environment [47]. 

Our results show that there are no major alterations in the secretion of wound healing-related cytokines by HFs after antibiotic treatments. However, there is a reduction in the secretion of IL-10, bFGF, and TNF-α after AMK and COL treatments in the case of hKTs, which is consistent with the fact that these treatments affect hKTs cell viability and proliferation. Therefore, we can state that 0.02 mg/mL CIP and 1 mg/mL MER are the highest non-cytotoxic concentrations for HFs and hKTs while 1.25 mg/mL AMK and 0.034 mg/mL COL do not affect HF viability and cytokine secretion, but they have an impact on hKTs. 

The concentrations tested are above the MIC of these antibiotics for *P. aeruginosa*. The MIC determination was carried out through three different methodologies, and these showed similar values (Table 2). These values agree with other studies [48,49,50,51]. However, when an infection occurs in severe injuries, a *P. aeruginosa* biofilm is formed. These bacterial communities are more difficult to eradicate compared to the planktonic forms of bacteria due to their intrinsic tolerance to antibiotics [11,52]. In this study, a calorimetric metabolic screening was conducted on biofilm formed on HF monolayers after treatment with different concentrations of antibiotics. Such calorimetric measurements were recently reported to accurately predict antibiotic activity [23,24,25]. Our results showed that the antibiotic concentrations needed to eradicate the bacterium attached to the HF monolayer are much higher than the concentrations killing the planktonic form of the bacterium. In other words, the bactericidal effects of antibiotics against the biofilm decrease compared with those on the planktonic forms. This is also reported by Marumo, K et al. regarding COL treatment of MDRP-YMD and ATCC27853 strains [53]. Focusing on the impact of antibiotics, it is observed how in the case of AMK, a range of concentrations from the highest non-cytotoxic to 500 µg/mL effectively eradicate the infection. However, for CIP, COL, and MER, the highest non-cytotoxic were unable to kill the bacterium although the bacterial growth after these treatments was significantly lower than the untreated biofilms. Therefore, for these antibiotics, concentrations above the highest non-cytotoxic are required. Importantly, when the highest non-cytotoxic concentrations of these antibiotics were combined with COL, all combinations eradicated the biofilm’s growth. This outcome highlights the need for combinations when applying these treatments to infected injuries. Antibiotic combinations with COL have been reported to be more effective against biofilms compared to monotherapies. Thus, Gómez-Juyent, J et al. reported that these combinations were the most appropriate treatments for biofilm-related infections [54] and Armengol, E et al. showed how COL enhances rifampicin’s antimicrobial activity in COL-resistant biofilms [55]. Wang Y et al. validated the effectiveness of the AMK–COL combination not only in vitro but also in an animal biofilm infection model [56]. Other antibiotic combinations have also been reported to be more effective than monotherapy [48]. The results obtained were consistent and provided valuable information, as quantitative measures of bacterial growth dynamics were obtained for long periods of time. In fact, calorimetric measurements of bacterial growth have been validated showing good concordance with the reference method for antibiotic combinations testing (checkerboard broth microdilution) against a large collection of MDR Gram-negative clinical isolates [57]. This is also supported by Kragh, K.M et al. who used microcalorimetric metabolic readout to predict whether combination treatment of MDR infections would have additive or synergistic effects [24]. 

This information provides critical information on the concentrations of these antibiotics that can be reached when administering topical treatments on skin lesions knowing if those concentrations have antibiofilm activity. This study highlights the efficacy of antibiotic combinations with COL. It should be noted that the highest noncytotoxic concentrations determined in this study could be higher in vivo thanks to the fact that skin cells are part of a three-dimensional environment that protects them. Importantly, these results allow us to gain insight into how these antibiotic treatments might affect wound management therapies such as the administration of autologous skin cell suspensions. Furthermore, it provides a base for the development of antibiotic-containing skin models. 

Finally, as a future perspective of this study, it would be convenient to establish the highest non-cytotoxic concentrations in the three-dimensional skin model and to analyze how a biofilm of *P. aeruginosa* grows in these models and how it behaves in the presence of these concentrations. This would allow us to establish the basis for future studies where the objective is the encapsulation of antibiotics and the development of skin models with antibiotic capacity. 

## Figures and Tables

**Table 1:** Concentrations of AMK, CIP, COL, and MER for *n* = 3 evaluation.

	HFs	hKTs
AMK	C2: 1.25 mg/mL	C2: 1.25 mg/mL
CIP	C4: 0.02 mg/mL	C4: 0.02 mg/mL
COL	C2: 0.034 mg/mL	C1: 0.017 mg/mL
MER	C1: 1 mg/mL	C1: 1 mg/mL

AMK, amikacin; CIP, ciprofloxacin; COL, colistin and MER, meropenem.

**Table 2:** MIC determination for AMK, CIP, COL, and MER against *P. aeruginosa* ATCC 27853.

	MIC ^1^ (µg/mL)	MIC ^2^ (µg/mL)	MIC ^3^ (µg/mL)
AMK	≤8	4	NA
CIP	0.25	0.19	NA
COL	NA	NA	2
MER	≤1	0.38	NA

AMK, amikacin; CIP, ciprofloxacin; COL, colistin; MER, meropenem; ^1^ MIC from MicroScan Walkaway system; ^2^ MIC from MIC Test Strip; ^3^ MIC from broth microdilution method; NA, not applicable.

## Data Availability

The datasets generated during and/or analyzed during the current study are available from the corresponding author upon reasonable request.

## Supplementary Materials

The following supporting information can be downloaded at: https://www.mdpi.com/article/10.3390/pharmaceutics16010117/s1, Figure S1: HFs proliferation after (**a**) AMK (0.625–12.5 mg/mL), (**b**) CIP (0.005–0.1 mg/mL), (**c**) COL (0.017–0.34 mg/mL), and (**d**) MER (0.05–1 mg/mL) treatments in a 14-day follow-up; Figure S2: hKTs proliferation after (**a**) AMK (0.625–12.5 mg/mL), (**b**) CIP (0.005–0.1 mg/mL), (**c**) COL (0.017–0.34 mg/mL), and (d) MER (0.05–1 mg/mL) treatments in a 14-day follow-up. Figure S3. Wound closure in HFs after antibiotic treatments. I. (**a**–**e**) Wound closure assay was used on HFs after 1.25 mg/mL AMK treatment at 0, 6, 12, 15, and 22 h after scratching, respectively. (**f**–**j**) HFs after 0.02 mg/mL CIP treatment at 0, 6, 12, 15, and 22 h after scratching, respectively. (**k**–**o**) HFs after 0.034 mg/mL COL treatment at 0, 6, 12, 15, and 22 h after scratching, respectively. (**p**–**t**) HFs after 1 mg/mL MER treatment at 0, 6, 12, 15, and 22 h after scratching, respectively. (**u**–**y**) Control at 0, 6, 12, 15, and 22 h after scratching, respectively. *n* = 3. Magnification 10×. Figure S4. Wound closure in HFs after antibiotic treatments. (**a**–**e**) Wound closure assay on hKTs after 1.25 mg/mL AMK treatment at 0, 12, 24, 30, and 34 h after scratching, respectively. (**f**–**j**) hKTs after 0.02 mg/mL CIP treatment at 0, 12, 24, 30, and 34 h after scratching, respectively. (**k**–**o**) hKTs after 0.017 mg/mL COL treatment at 0, 12, 24, 30, and 34 h after scratching, respectively. (**p**–**t**) hKTs after 1 mg/mL MER treatment at 0, 12, 24, 30, and 34 h after scratching, respectively. (**u**–**y**) Control at 0, 12, 24, 30, and 34 h after scratching, respectively. *n* = 3. Magnification 10×. Table S1: The concentration levels of IL-10 in HF supernatants for each treatment and control measured at days 3, 7, 10, and 14; *n*=3, the values are expressed as mean ± SEM (pg/mL).; Table S2: The concentration levels of bFGF in the supernatants of HF for each treatment and control measured at days 3, 7, 10, and 14; *n* = 3, the values are expressed as mean ± SEM (pg/mL); Table S3: The concentration levels of TNF-α in the supernatants of HF for each treatment and control measured at days 3, 7, 10, and 14; *n* = 3, the values are expressed as mean ± SEM (pg/mL); Table S4: The concentration levels of IL-6 in the supernatants of HF for each treatment and control measured at days 3, 7, 10, and 14; *n* = 3, the values are expressed as mean ± SEM (pg/mL); Table S5: The concentration levels of IL-10 in the supernatants of hKTs for each treatment and control measured at days 3, 7, 10, and 14; *n* = 3, the values are expressed as mean ± SEM (pg/mL); Table S6: The concentration levels of bFGF in the supernatants of hKTs for each treatment and control measured at days 3, 7, 10, and 14; *n* = 3, the values are expressed as mean ± SEM (pg/mL); Table S7: The concentration levels of TNF-α in the supernatants of hKTs for each treatment and control measured at days 3, 7, 10, and 14; *n* = 3, the values are expressed as mean ± SEM (pg/mL); Table S8: The concentration levels of IL-6 in the supernatants of hKTs for each treatment and control measured at days 3, 7, 10, and 14; *n* = 3, the values are expressed as mean ± SEM (pg/mL).
