# Supplementary material for: Antibiotics against Pseudomonas aeruginosa on Human Skin Cell Lines: Determination of the Highest Non-Cytotoxic Concentrations with Antibiofilm Capacity for Wound Healing Strategies
Source: Pharmaceutics. 2024 Jan 17;16(1):117. doi: 10.3390/pharmaceutics16010117 (PMC10818945; doi:10.3390/pharmaceutics16010117)
Supplement: Supplementary file 1 [file pharmaceutics-16-00117-s001.zip › pharmaceutics-2805185-supplementary.pdf]

# Supplementary Materials

**Article Title:** Antibiotics against *Pseudomonas aeruginosa* on Human Skin Cell Lines: Determination of the Highest Non-Cytotoxic Concentrations with Antibiofilm Capacity for Wound Healing Strategies.

**Authors and affiliations:**

María I. Quiñones-Vico[1,2,3,4,5], Ana Fernández-González[1,2,3*], Ana Ubago-Rodríguez[1,2,3], Kirsten Moll[6], Anna Norrby-Teglund[6], Mattias Svensson[6], José Gutiérrez-Fernández[7], Jesús M. Torres[5] and Salvador Arias-Santiago[1,2,3,4,8].

[1]Cell Production and Tissue Engineering Unit, Virgen de las Nieves University Hospital, 18014 Granada, Spain

[2]Biosanitary Institute of Granada (ibs.GRANADA), 18014 Granada, Spain

[3]Andalusian Network of Design and Translation of Advanced Therapies, 41092 Seville, Spain

[4]Dermatology Department, School of Medicine, University of Granada, 18014 Granada, Spain

[5]Biochemistry, Molecular Biology III and Immunology Department, University of Granada, 18071, Spain.

[6]Center for Infectious Medicine, Karolinska Institutet, Karolinska University Hospital Huddinge, Stockholm, Sweden

[7]Department of Microbiology, Virgen de las Nieves University Hospital, 18014 Granada, Spain

[8]Dermatology Department, Virgen de las Nieves University Hospital, 18014 Granada, Spain

Correspondence: ana.fernandez.gonzalez@juntadeandalucia.es

***Preliminary Evaluation.***

*High Concentration of Antibiotics Cause a Reduction in Cell Proliferation in Skin Cell Lines.*

For HFs, 1.25 mg/mL AMK, 0.02 mg/mL CIP, 0.034 mg/mL COL and 1 mg/mL MER were the concentrations maintaining a cell proliferation level comparable to the control (Figure S1). For hKTs, 1.25 mg/mL AMK, 0.02 mg/mL CIP, 0.017 mg/mL COL and 1 mg/mL MER (Figure S2) were chosen for the following evaluation. It should be note that cell proliferation after AMK and COL treatments were notably reduced when compared to the control group.

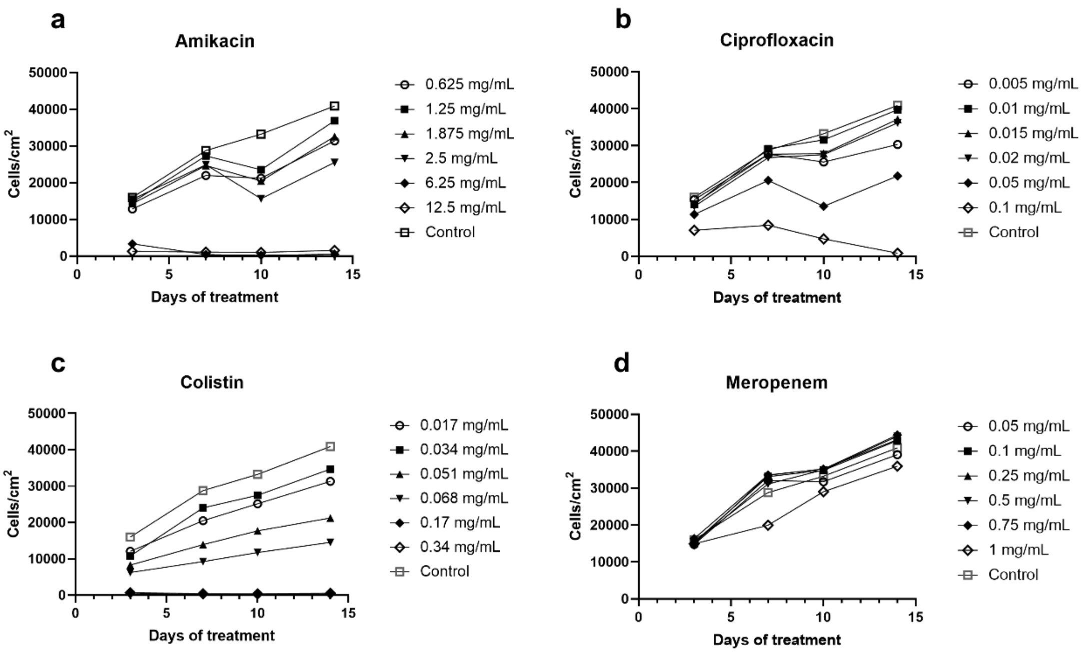


**Figure S1.** HFs proliferation after (**a**) AMK (0.625 - 12.5 mg/mL), (**b**) CIP (0.005 - 0.1 mg/mL), (**c**) COL (0.017 - 0.34 mg/mL) and (**d**) MER (0.05 - 1 mg/mL) treatments in a 14-day follow-up.

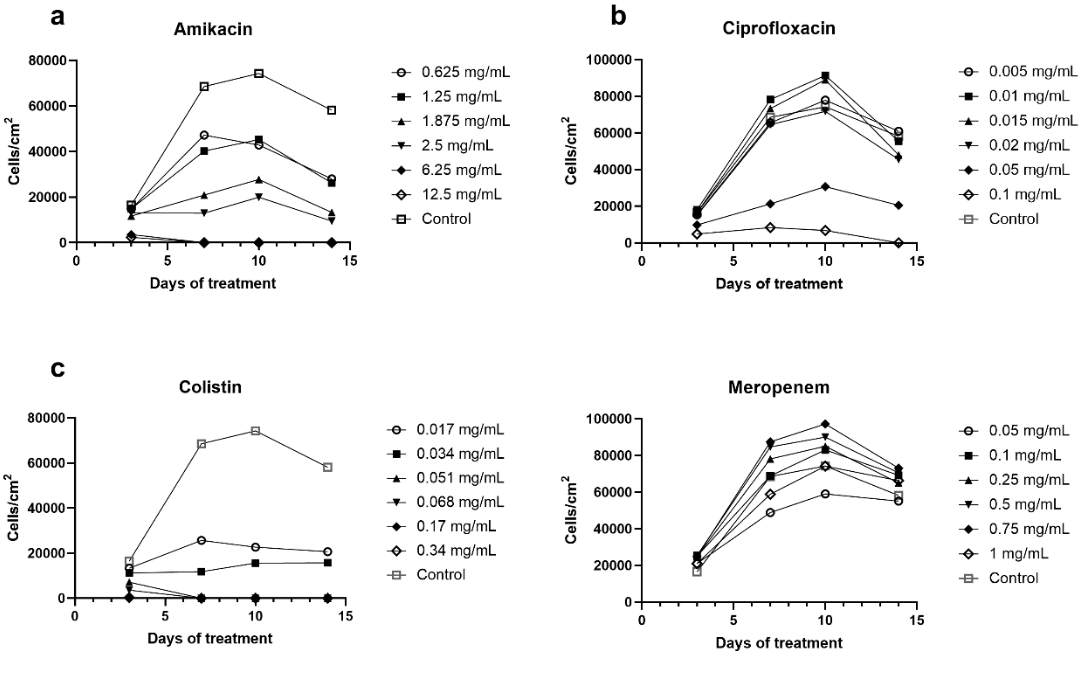


**Figure S2.** hKTs proliferation after (**a**) AMK (0.625 - 12.5 mg/mL), (**b**) CIP (0.005 - 0.1 mg/mL), (**c**) COL (0.017 - 0.34 mg/mL) and (**d**) MER (0.05 - 1 mg/mL) treatments in a 14-day follow-up.

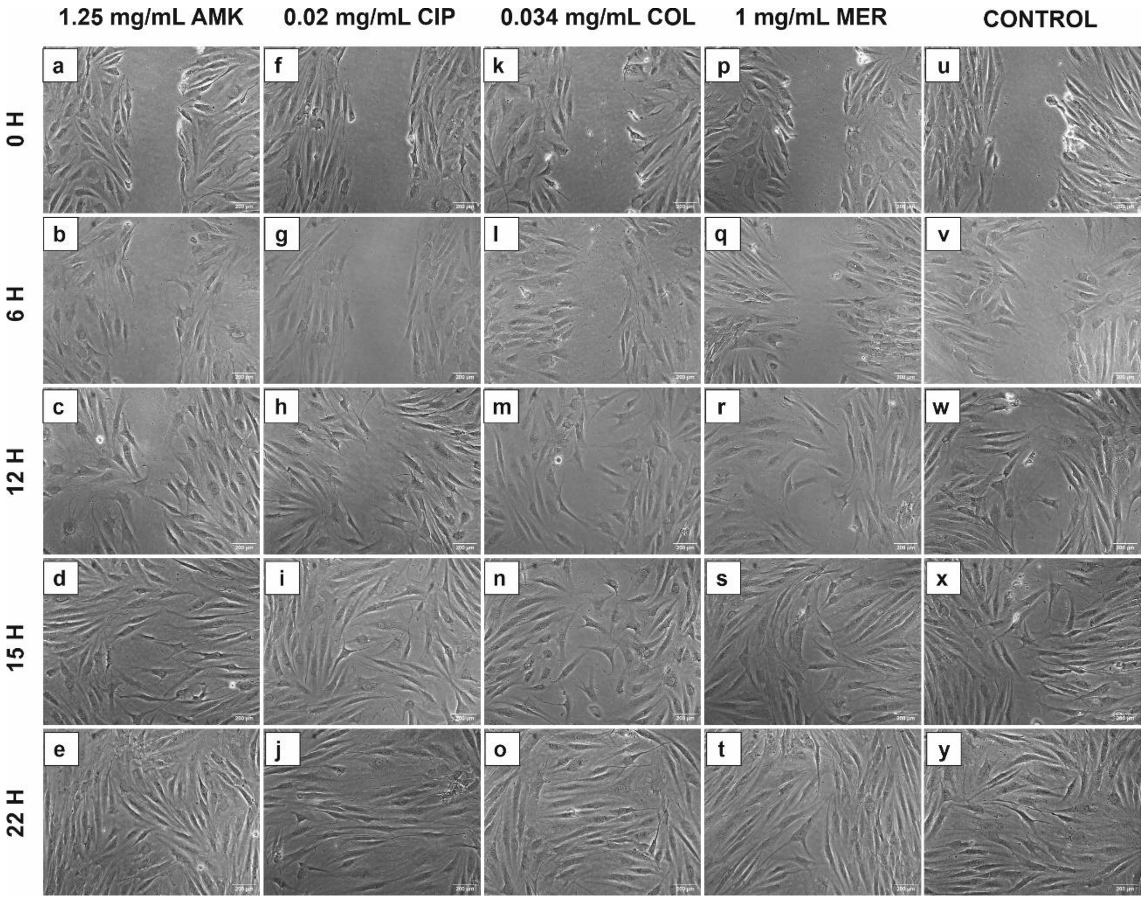


**Figure S3.** Wound closure in HFs after antibiotic treatments. I. (a-e) Wound closure assay was used on HFs after 1.25 mg/mL AMK treatment at 0,6,12, 15 and 22 h after scratching, respectively. (f–j) HFs after 0.02 mg/mL CIP treatment at 0,6,12, 15 and 22 h after scratching, respectively. (k-o) HFs after 0.034 mg/mL COL treatment at 0,6,12, 15 and 22 h after scratching, respectively. (p-t) HFs after 1 mg/mL MER treatment at 0,6,12, 15 and 22 h after scratching, respectively. (u-y) Control at 0, 6, 12, 15 and 22 h after scratching, respectively. n = 3. Magnification 10x.

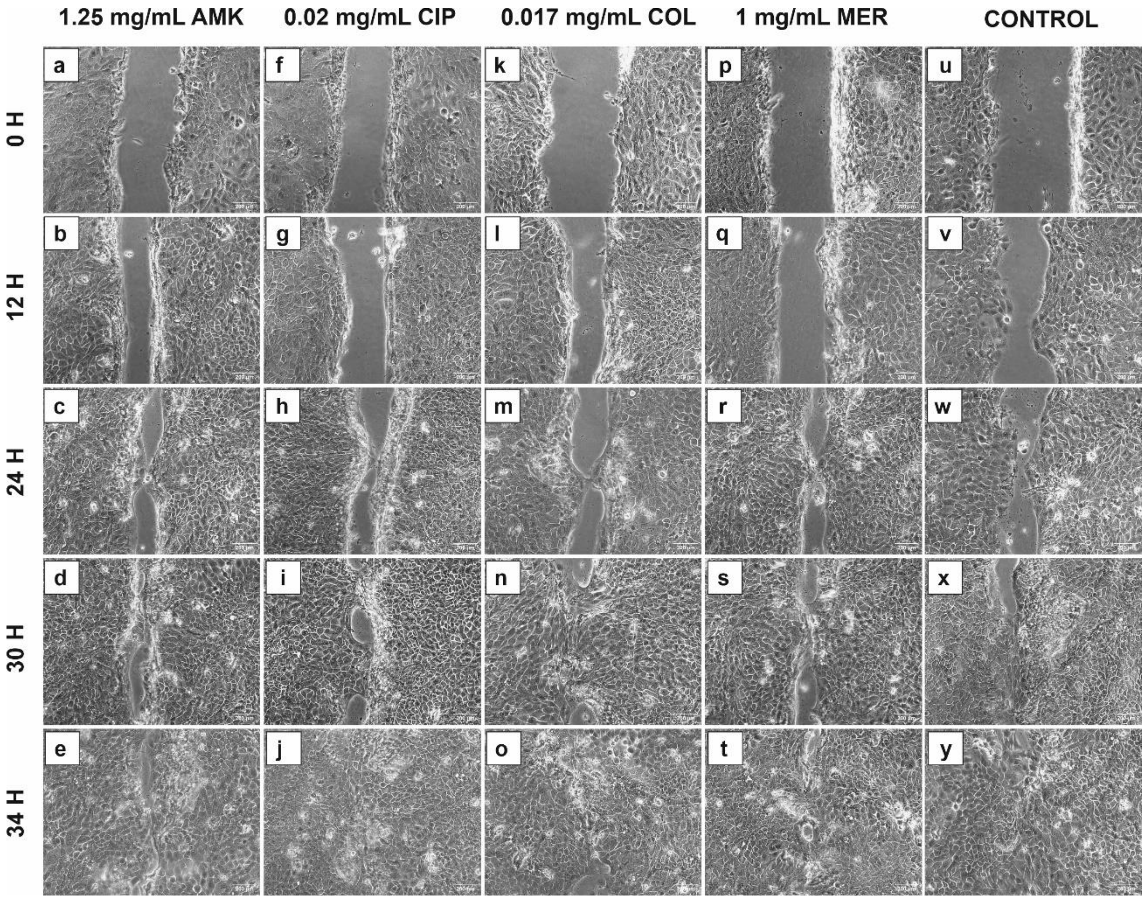


**Figure S4.** Wound closure in HFs after antibiotic treatments. (a-e) Wound closure assay on hKTs after 1.25 mg/mL AMK treatment at 0, 12, 24, 30 and 34 h after scratching, respectively. (f–j) hKTs after 0.02 mg/mL CIP treatment at 0, 12, 24, 30 and 34 h after scratching, respectively. (k-o) hKTs after 0.017 mg/mL COL treatment at 0, 12, 24, 30 and 34 h after scratching, respectively. (p-t) hKTs after 1 mg/mL MER treatment at 0, 12, 24, 30 and 34 h after scratching, respectively. (u-y) Control at 0, 12, 24, 30 and 34 h after scratching, respectively. n = 3. Magnification 10x.

**Table S1.** IL-10 concentration levels in HF supernatants for each treatment and control at days 3, 7, 10 and 14; n = 3, values expressed as mean (pg/mL).

| *Treatment* | *D3* | *D7* | *D10* | *D14* |
|---|---|---|---|---|
| *Amikacin 1.25 mg/mL* | 12.76 ± 1.99 | 14.97 ± 0.28 | 17.70 ± 0.22 | 15.94 ± 3.86 |
| *Ciprofloxacin 0.02 mg/mL* | 11.31 ± 0.76 | 15.97 ± 1.56 | 17.08 ± 0.57 | 18.19 ± 0.03 |
| *Colistin 0.034 mg/mL* | 11.03 ± 0.54 | 12.47 ± 0.11 | 12.28 ± 1.05 | 13.27 ± 0.45 |
| *Meropenem 1 mg/mL* | 10.69 ± 0.37 | 10.34 ± 1.73 | 12.33 ± 0.36 | 13.95 ± 1.19 |
| *Control* | 14.43 ± 0.08 | 17.53 ± 0.11 | 20.34 ± 0.82 | 20.20 ± 0.79 |

**Table S2.** bFGF concentration levels in HF supernatants for each treatment and control at days 3, 7, 10 and 14; n = 3, values expressed as mean (pg/mL).

| *Treatment* | *D3* | *D7* | *D10* | *D14* |
|---|---|---|---|---|
| *Amikacin 1.25 mg/mL* | 181 ± 6.67 | 224.33 ± 23.33 | 229.33 ± 21.66 | 274.33 ± 50 |
| *Ciprofloxacin 0.02 mg/mL* | 206 ± 48.33 | 294.33 ± 43.33 | 236 ± 41.67 | 294.33 ± 3.33 |
| *Colistin 0.034 mg/mL* | 197.67 ± 30 | 204.33 ± 33.33 | 212.66 ± 28.33 | 214.33 ± 16.66 |
| *Meropenem 1 mg/mL* | 192.67 ± 5 | 211 ± 30 | 236 ± 48.33 | 237.66 ± 13.33 |
| *Control* | 279.33 ± 25 | 292.66 ± 31.66 | 366 ± 38.33 | 414.33 ± 20 |

**Table S3.** TNF-$\alpha$ concentration levels in HF supernatants for each treatment and control at days 3, 7, 10 and 14; n = 3, values expressed as mean (pg/mL).

| *Treatment* | *D3* | *D7* | *D10* | *D14* |
|---|---|---|---|---|
| *Amikacin 1.25 mg/mL* | 692.18 ± 38.75 | 760.93 ± 7.5 | 888.43 ± 70 | 643.43 ± 172.5 |
| *Ciprofloxacin 0.02 mg/mL* | 617.18 ± 13.75 | 617.18 ± 13.75 | 800.93 ± 107.5 | 697.18 ± 3.75 |
| *Colistin 0.034 mg/mL* | 505.93 ± 7.5 | 485.93 ± 85 | 466.43 ± 23 | 558.43 ± 62.5 |
| *Meropenem 1 mg/mL* | 484.68 ± 23.75 | 617.18 ± 46.25 | 643.43 ± 77.5 | 688.43 ± 45 |
| *Control* | 673.43 ± 2.5 | 697.18 ± 36.25 | 963.43 ± 97.5 | 742.18 ± 1.25 |

**Table S4.** IL-6 concentration levels in HF supernatants for each treatment and control at days 3, 7, 10 and 14; n = 3, values expressed as mean (pg/mL).

| *Treatment* | *D3* | *D7* | *D10* | *D14* |
|---|---|---|---|---|
| *Amikacin 1.25 mg/mL* | 4348.89 ± 2233.33 | 1607.22 ± 380.55 | 1654.44 ± 133.33 | 3293.33 ± 1983.33 |
| *Ciprofloxacin 0.02 mg/mL* | 2871.11 ± 1433.33 | 1782.22 ± 455.55 | 2185 ± 102.78 | 2773.89 ± 1002.78 |
| *Colistin 0.034 mg/mL* | 3276.67 ± 1300 | 1679.44 ± 575 | 1754.44 ± 372.25 | 2385 ± 1163.89 |
| *Meropenem 1 mg/mL* | 2346.11 ± 902.78 | 1665.55 ± 516.65 | 1271.11 ± 77.78 | 1482.22 ± 361.11 |
| *Control* | 3371.11 ± 1783.33 | 1685 ± 191.67 | 1679.44 ± 202.77 | 2193.33 ± 672.25 |

**Table S5.** IL-10 concentration levels in hKTs cells supernatants for each treatment and control at days 3, 7, 10 and 14; n = 3, values expressed as mean (pg/mL).

| *Treatment* | *D3* | *D7* | *D10* | *D14* |
|---|---|---|---|---|
| *Amikacin 1.25 mg/mL* | 5.18 ± 0.15 | 4.67 ± 0.03 | 4.78 ± 0.26 | 5.07 ± 0.66 |
| *Ciprofloxacin 0.02 mg/mL* | 4.78 ± 0.37 | 7.22 ± 0.54 | 8.82 ± 0.77 | 10.49 ± 0.68 |
| *Colistin 0.017 mg/mL* | 3.10 ± 0.17 | 3.70 ± 0.03 | 3.33 ± 0.06 | 2.74 ± 0.15 |
| *Meropenem 1 mg/mL* | 4.21 ± 1.45 | 5.07 ± 0.09 | 7.45 ± 1.39 | 7.71 ± 0.29 |
| *Control* | 4.49 ± 0.54 | 8.76 ± 1.96 | 9.81 ± 1.65 | 15.09 ± 1.42 |

**Table S6.** bFGF concentration levels in hKTs cells supernatants for each treatment and control at days 3, 7, 10 and 14; n = 3, values expressed as mean (pg/mL).

| *Treatment* | *D3* | *D7* | *D10* | *D14* |
|---|---|---|---|---|
| *Amikacin 1.25 mg/mL* | 126 ± 8.33 | 116 ± 5 | 124.33 ± 0 | 129.33 ± 1.66 |
| *Ciprofloxacin 0.02 mg/mL* | 122.66 ± 1.66 | 134.33 ± 13.33 | 219.33 ± 5 | 251 ± 30 |
| *Colistin 0.017 mg/mL* | 142.66 ± 21.66 | 129.33 ± 1.66 | 127.66 ± 3.33 | 122.67 ± 5 |
| *Meropenem 1 mg/mL* | 134.33 ± 10 | 151 ± 6.67 | 207.66 ± 3.33 | 234.33 ± 23.33 |
| *Control* | 136 ± 8.33 | 202.67 ± 15 | 309.33 ± 15 | 556 ± 1.67 |

**Table S7.** TNF-$\alpha$ concentration levels in hKTs cells supernatants for each treatment and control at days 3, 7, 10 and 14; n = 3, values expressed as mean (pg/mL).

| *Treatment* | *D3* | *D7* | *D10* | *D14* |
|---|---|---|---|---|
| *Amikacin 1.25 mg/mL* | 279.68 ± 26.25 | 293.43 ± 10 | 350.93 ± 7.5 | 299.68 ± 26.25 |
| *Ciprofloxacin 0.02 mg/mL* | 267.18 ± 6.25 | 395.93 ± 15 | 570.93 ± 62.5 | 519.68 ± 56.25 |
| *Colistin 0.017 mg/mL* | 240.93 ± 15 | 284.68 ± 18.75 | 382.18 ± 28.75 | 268.43 ± 15 |
| *Meropenem 1 mg/mL* | 242.18 ± 6.25 | 374.68 ± 23.75 | 454.68 ± 28.75 | 514.68 ± 36.25 |
| *Control* | 299.68 ± 18.75 | 507.18 ± 68.75 | 629.68 ± 8.75 | 678.43 ± 15 |

**Table S8.** IL-6 concentration levels in hKTs supernatants for each treatment and control at days 3, 7, 10 and 14; n = 3, values expressed as mean (pg/mL).

| *Treatment* | *D3* | *D7* | *D10* | *D14* |
|---|---|---|---|---|
| *Amikacin 1.25 mg/mL* | 876.7 ± 11.1 | 862.8 ± 2.8 | 960 ± 138.9 | 823.9 ± 2.8 |
| *Ciprofloxacin 0.02 mg/mL* | 843.35 ± 22.25 | 1023.85 ± 30.55 | 1154.45 ± 33.35 | 1040.55 ± 47.25 |
| *Colistin 0.017 mg/mL* | 829.45 ± 2.75 | 996.1 ± 13.9 | 1010 ± 22.2 | 1018.35 ± 8.35 |
| *Meropenem 1 mg/mL* | 782.25 ± 5.55 | 1162.75 ± 108.35 | 1312.75 ± 91.65 | 757.2 ± 15.9 |
| *Control* | 854.4 ± 0 | 1271.1 ± 27.8 | 1110 ± 72.2 | 1032.2 ± 0 |
